# Chapter 12: Human Microbiome Analysis

**DOI:** 10.1371/journal.pcbi.1002808

**Published:** 2012-12-27

**Authors:** Xochitl C. Morgan, Curtis Huttenhower

**Affiliations:** 1 Department of Biostatistics, Harvard School of Public Health, Boston, Massachusetts, United States of America; 2 The Broad Institute of MIT and Harvard, Cambridge, Massachusetts, United States of America; Whitehead Institute, United States of America; University of Maryland, Baltimore County, United States of America

## Abstract

Humans are essentially sterile during gestation, but during and after birth, every body surface, including the skin, mouth, and gut, becomes host to an enormous variety of microbes, bacterial, archaeal, fungal, and viral. Under normal circumstances, these microbes help us to digest our food and to maintain our immune systems, but dysfunction of the human microbiota has been linked to conditions ranging from inflammatory bowel disease to antibiotic-resistant infections. Modern high-throughput sequencing and bioinformatic tools provide a powerful means of understanding the contribution of the human microbiome to health and its potential as a target for therapeutic interventions. This chapter will first discuss the historical origins of microbiome studies and methods for determining the ecological diversity of a microbial community. Next, it will introduce shotgun sequencing technologies such as metagenomics and metatranscriptomics, the computational challenges and methods associated with these data, and how they enable microbiome analysis. Finally, it will conclude with examples of the functional genomics of the human microbiome and its influences upon health and disease.

**What to Learn in This Chapter**

- An overview of the analysis of microbial communities
- Understanding the human microbiome from phylogenetic and functional perspectives
- Methods and tools for calculating taxonomic and phylogenetic diversity
- Metagenomic assembly and pathway analysis
- The impact of the microbiome on its host

This article is part of the “Translational Bioinformatics” collection for *PLOS Computational Biology*.

## 1. Introduction

The question of what it means to be human is more often encountered in metaphysics than in bioinformatics, but it is surprisingly relevant when studying the human microbiome. We are born consisting only of our own eukaryotic human cells, but over the first several years of life, our skin surface, oral cavity, and gut are colonized by a tremendous diversity of bacteria, archaea, fungi, and viruses. The community formed by this complement of cells is called the human microbiome; it contains almost ten times as many cells as are in the rest of our bodies and accounts for several pounds of body weight and orders of magnitude more genes than are contained in the human genome [1], [2]. Under normal circumstances, these microbes are commensal, helping to digest our food and to maintain our immune systems. Although the human microbiome has long been known to influence human health and disease [1], we have only recently begun to appreciate the breadth of its involvement. This is almost entirely due to the recent ability of high-throughput sequencing to provide an efficient and cost-effective tool for investigating the members of a microbial community and how they change. Thus, dysfunctions of the human microbiota are increasingly being linked to disease ranging from inflammatory bowel disease to diabetes to antibiotic-resistant infection, and the potential of the human microbiome as an early detection biomarker and target for therapeutic intervention is a vibrant area of current research.

## 2. A Brief History of Microbiome Studies

Historically, members of a microbial community were identified *in situ* by stains that targeted their physiological characteristics, such as the Gram stain [3]. These could distinguish many broad clades of bacteria but were non-specific at lower taxonomic levels. Thus, microbiology was almost entirely culture-dependent; it was necessary to grow an organism in the lab in order to study it. Specific microbial species were detected by plating samples on specialized media selective for the growth of that organism, or they were identified by features such as the morphological characteristics of colonies, their growth on different media, and metabolic production or consumption. This approach limited the range of organisms that could be detected to those that would actively grow in laboratory culture, and it led the close study of easily-grown, now-familiar model organisms such as *Escherichia coli*. However, *E. coli* as a taxonomic unit accounts for at most 5% of the microbes occupying the typical human gut [2]. The vast majority of microbial species have never been grown in the laboratory, and options for studying and quantifying the uncultured were severely limited until the development of DNA-based culture-independent methods in the 1980s [4].

**Figure 1:**
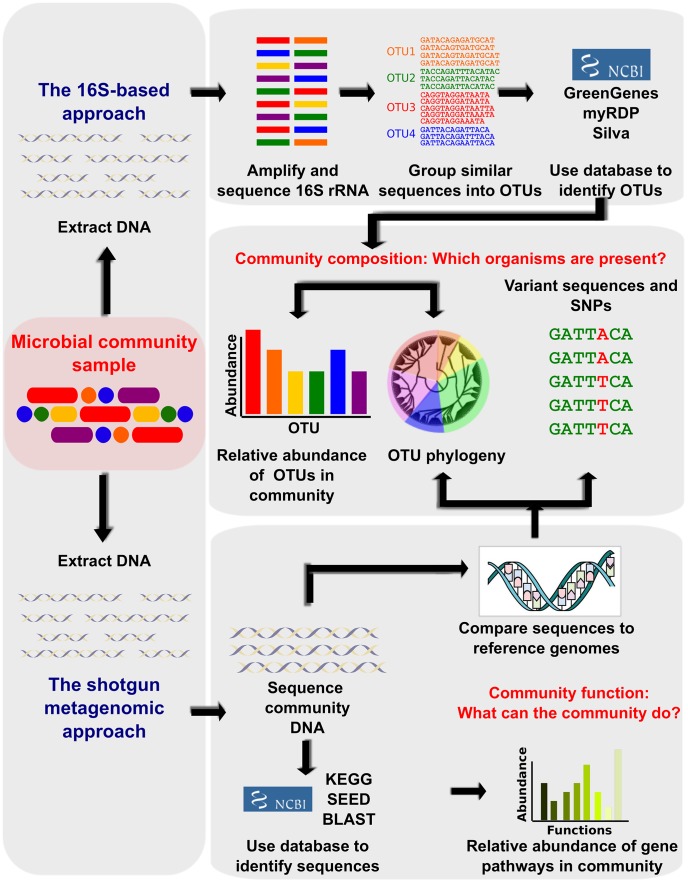
Bioinformatic methods for functional metagenomics. Studies that aim to define the composition and function of uncultured microbial communities are often referred to collectively as “metagenomic,” although this refers more specifically to particular sequencing-based assays. First, community DNA is extracted from a sample, typically uncultured, containing multiple microbial members. The bacterial taxa present in the community are most frequently defined by amplifying the 16S rRNA gene and sequencing it. Highly similar sequences are grouped into Operational Taxonomic Units (OTUs), which can be compared to 16S databases such as Silva [16], Green Genes [14], and RDP [15] to identify them as precisely as possible. The community can be described in terms of which OTUs are present, their relative abundance, and/or their phylogenetic relationships. An alternate method of identifying community taxa is to directly metagenomically sequence community DNA and compare it to reference genomes or gene catalogs. This is more expensive but provides improved taxonomic resolution and allows observation of single nucleotide polymorphisms (SNPs) and other variant sequences. The functional capabilities of the community can also be determined by comparing the sequences to functional databases (e.g. KEGG [170] or SEED [171]). This allows the community to be described as relative abundances of its genes and pathways. Figure adapted from [172].

Culture-independent techniques, which analyze the DNA extracted directly from a sample rather than from individually cultured microbes, allow us to investigate several aspects of microbial communities (Figure 1). These include taxonomic diversity, such as how many of which microbes are present in a community, and functional metagenomics, which attempts to describe which biological tasks the members of a community can or do carry out. The earliest DNA-based methods probed extracted community DNA for genes of interest by hybridization, or amplified specifically-targeted genes by PCR prior to sequencing. These studies were typically able to describe diversity at a broad level, or detect the presence or absence of individual biochemical functions, but with few details in either case.

One of the earliest targeted metagenomic assays for studying uncultured communities without prior DNA extraction was fluorescent *in situ* hybridization (FISH), in which fluorescently-labeled, specific oligonuclotide probes for marker genes are hybridized to a microbial community [5]. FISH probes can be targeted to almost any level of taxonomy from species to phylum. Although FISH was initially limited to the 16S rRNA marker gene and thus to diversity studies, it has since been expanded to functional gene probes that can be used to identify specific enzymes in communities [6]. However, it remains a primarily low-throughput, imaging-based technology.

To investigate microbial communities efficiently at scale, almost all current studies employ high-throughput DNA sequencing, increasingly in combination with other genome-scale platforms such as proteomics or metabolomics. Although DNA sequencing has existed since the 1970s [7], [8], it was historically quite expensive; sequencing environmental DNA further required the additional time and expense of clone library construction. It was not until the 2005 advent of next-generation high-throughput sequencing [9] that it became economically feasible for most scientists to sequence the DNA of an entire environmental sample, and metagenomic studies have since become increasingly common.

## 3. Taxonomic Diversity

### 3.1 The 16S rRNA Marker Gene

Like a metazoan, a microbial community consists fundamentally of a collection of individual cells, each carrying a distinct complement of genomic DNA. Communities, however, obviously differ from multicellular organisms in that their component cells may or may not carry identical genomes, although substantial subsets of these cells are typically assumed to be clonal. One can thus assign a frequency to each distinct genome within the community describing either the absolute number of cells in which it is carried or their relative abundance within the population. As it is impractical to fully sequence every genome in every cell (a statement that should remain safely true no matter how cheap high-throughput sequencing becomes), microbial ecology has defined a number of molecular markers that (more or less) uniquely tag distinct genomes. Just as the make, model, and year of a car identify its components without the need to meticulously inspect the entirety of every such car, a marker is a DNA sequence that identifies the genome that contains it, without the need to sequence the entire genome.

Although different markers can be chosen for analyzing different populations, several properties are desirable for a good marker. A marker should be present in every member of a population, should differ only and always between individuals with distinct genomes, and, ideally, should differ proportionally to the evolutionary distance between distinct genomes. Several such markers have been defined, including ribosomal protein subunits, elongation factors, and RNA polymerase subunits [10], but by far the most ubiquitous (and historically significant [11]) is the small or 16S ribosomal RNA subunit gene [12]. This 1.5 Kbp gene is commonly referred to as the 16S rRNA (after transcription) or sometimes rDNA; it satisfies the criteria of a marker by containing both highly conserved, ubiquitous sequences and regions that vary with greater or lesser frequency over evolutionary time. It is relatively cheap and simple to sequence only the 16S sequences from a microbiome [13], thus describing the population as a set of 16S sequences and the number of times each was detected. Sequences assayed in this manner have been characterized for a wide range of cultured species and environmental isolates; these are stored and can be automatically matched against several databases including GreenGenes [14], the Ribosomal Database Project [15], and Silva [16].

### 3.2 Binning 16S rRNA Sequences into OTUs

A bioinformatic challenge that arises immediately in the analysis of rRNA genes is the precise definition of a “unique” sequence. Although much of the 16S rRNA gene is highly conserved, several of the sequenced regions are variable or hypervariable, so small numbers of base pairs can change in a very short period of evolutionary time [17]. Horizontal transfer, multicopy or ambiguous rDNA markers, and other confounding factors do, however, blur the biological meaning of “species” as well as our ability to resolve them technically [17]. Finally, because 16S regions are typically sequenced using only a single pass, there is a fair chance that they will thus contain at least one sequencing error. This means that requiring tags to be 100% identical will be extremely conservative and treat essentially clonal genomes as different organisms. Some degree of sequence divergence is typically allowed - 95%, 97%, or 99% are sequence similarity cutoffs often used in practice [18] - and the resulting cluster of nearly-identical tags (and thus assumedly identical genomes) is referred to as an Operational Taxonomic Unit (OTU) or sometimes phylotype. OTUs take the place of “species” in many microbiome diversity analyses because named species genomes are often unavailable for particular marker sequences. The assignment of sequences to OTUs is referred to as binning, and it can be performed by A) unsupervised clustering of similar sequences [19], B) phylogenetic models incorporating mutation rates and evolutionary relationships [20], or C) supervised methods that directly assign sequences to taxonomic bins based on labeled training data [21] (which also applies to whole-genome shotgun sequences; see below).

The binning process allows a community to be analyzed in terms of discrete bins or OTUs, opening up a range of computationally tractable representations for biological analysis. If each OTU is treated as a distinct category, or each 16S sequence is binned into a named phylum or other taxonomic category, a pool of microbiome sequences can be represented as a histogram of bin counts [22]. Alternately, this histogram can be binarized into presence/absence calls for each bin across a collection of related samples. Because diverse, general OTUs will always be present in related communities, and overly-specific OTUs may not appear outside of their sample of origin, the latter approach is typically most useful for low-complexity microbiomes or OTUs at an appropriately tuned level of specificity. Bioinformaticians studying 16S sequences must choose whether to analyze a collection of taxonomically-binned microbiomes as a set of abundance histograms, or as a set of binary presence/absence vectors. However, either representation can be used as input to decomposition methods such as Principle Components Analysis or Canonical Correlation Analysis [23] to determine which OTUs represent the most significant sources of population variance and/or correlate with community metadata such as temperature, pH, or clinical features [24], [25].

### 3.3 Measuring Population Diversity

An important concept when dealing with OTUs or other taxonomic bins is that of population diversity, the number of distinct bins in a sample or in the originating population. This is of critical importance in human health, since a number of disease conditions have been shown to correlate with decreased microbiome diversity, presumably as one or a few microbes overgrow during immune or nutrient imbalance in a process not unlike an algal bloom [26]. Intriguingly, recent results have also shown that essentially no bacterial clades are widely and consistently shared among the human microbiome [2]. Many organisms are abundant in some individuals, and many organisms are prevalent among most individuals, but none are universal. Although they can vary over time and share some similarity with some individuals, our intestinal contents appear to be highly personalized when considered in terms of microbial presence, absence, and abundance.

**Figure 2:**
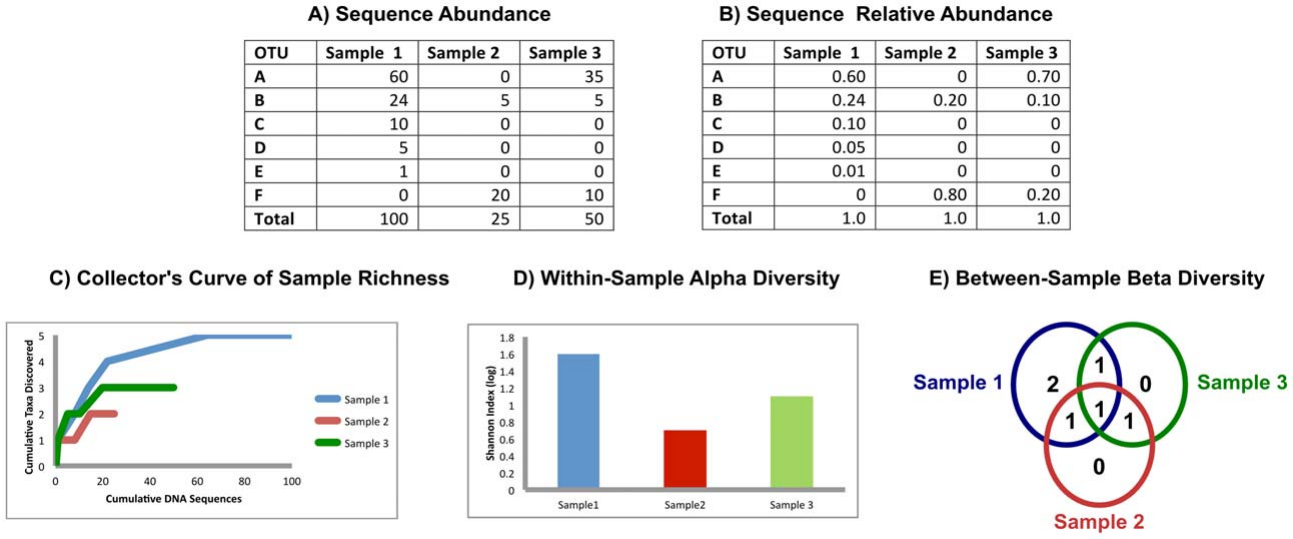
Ecological representations of microbial communities: collector's curves, alpha, and beta diversity. These examples describe the A) sequence counts and B) relative abundances of six taxa (A, B, C, D, E, and F) detected in three samples. C) A collector's curve, typically generated using a richness estimator such as Chao1 [28] or ACE [29], approximates the relationship between the number of sequences drawn from each sample and the number of taxa expected to be present based on detected abundances. D) Alpha diversity captures both the organismal richness of a sample and the evenness of the organisms' abundance distribution. Here, alpha diversity is defined by the Shannon index [32], 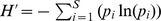, where *p_i_* is the relative abundance of taxon *i*, although many other alpha diversity indices may be employed. E) Beta diversity represents the similarity (or difference) in organismal composition between samples. In this example, it can be simplistically defined by the equation 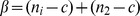, where n_1_ and n_2_ are the number of taxa in samples 1 and 2, respectively, and *c* is the number of shared taxa, but again many metrics such as Bray-Curtis [34] or UniFrac [24] are commonly employed.

Two mathematically well-defined questions arise when quantifying population diversity (Figure 2): given that *x* bins have been observed in a sample of size *y* from a population of size *z*, how many bins are expected to exist in the population; or, given that *x* bins exist in a population of size *z*, how big must the sample size *y* be to observe all of them at least once? In other words, “If I've sequenced some amount of diversity, how much more exists in my microbiome?” and, “How much do I need to sequence to completely characterize my microbiome?” The latter is known as the Coupon Collector's Problem, as identical questions can be asked if a cereal manufacturer has randomly hidden one of several different possible prize coupons in each box of cereal [27]. Within a community, several estimators including the Chao1 [28], Abundance-based Coverage Estimator (ACE) [29], and Jackknife [30] measures exist for calculating alpha diversity, the number (richness) and distribution (evenness) of taxa expected within a single population. These give rise to figures known as collector's or rarefaction curves, since increasing numbers of sequenced taxa allow increasingly precise estimates of total population diversity [31]. Additionally, when comparing multiple populations, beta diversity measures including absolute or relative overlap describe how many taxa are shared between them (Figure 2). An alpha diversity measure thus acts like a summary statistic of a single population, while a beta diversity measure acts like a similarity score between populations, allowing analysis by sample clustering or, again, by dimensionality reductions such as PCA [20]. Alpha diversity is often quantified by the Shannon Index [32], 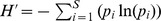, or the Simpson Index [33], 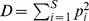, where 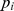 is the fraction of total species comprised by species *i*. Beta diversity can be measured by simple taxa overlap or quantified by the Bray-Curtis dissimilarity [34], 
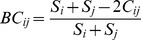
, where *S_i_* and *S_j_* are the number of species in populations *i* and *j*, and *C_ij_* is the total number of species at the location with the fewest species. Like similarity measures in expression array analysis, many alpha- and beta-diversity measures have been developed that each reveal slightly different aspects of community ecology.

Alternatively, the diversity within or among communities can be analyzed in terms of its phylogenetic distribution rather than by isolating discrete bins. This method of quantifying community diversity describes it in terms of the total breadth or depth of the phylogenetic branches spanned by a microbiome (or shared among two or more). For example, consider a collection of *n* highly-related 16S sequences. These might be treated either as one OTU or as *n* distinct taxa, depending on how finely they are binned, but a phylogenetic analysis will consider them to span a small evolutionary distance no matter how large *n* becomes. Conversely, two highly-divergent binned OTUs are typically no different than two similar OTUs, but a phylogenetic method would score them as spanning a large evolutionary distance. OTU-based and phylogenetic methods tend to be complementary, in that each will reveal different aspects of community structure. OTUs are highly sensitive to the specific means by which taxa are binned, for example, whereas phylogenetic measures are sensitive to the method of tree construction. Like the OTU-based diversity estimators discussed above, several standard metrics such as UniFrac [20] exist for quantifying phylogenetic diversity, and these can be treated as single-sample descriptors or as multiple-sample similarity measures.

It is critically important in any microbiome richness analysis to account for the contribution that technical noise will make to apparent diversity. As a simple example, consider that a single base pair error in a 100 bp sequence read will create a new OTU at the 99% similarity threshold. Apparent diversity can thus be dramatically modified by the choice of marker gene, the region within it that is sequenced, the biochemical marker extraction and amplification processes, and the read length and noise characteristics of the sequencing platform. Accounting for such errors computationally continues to be a fruitful area of research, particularly as 454-based technologies have transitioned to the Illumina platform, as current solutions can discard all but the highest-quality sequence regions [18]. A major confound in many early molecular richness analyses was the abundance of chimeric sequences, or reads in which two unique marker sequences (typically 16S regions) adhere during the amplification process, creating an apparently novel taxon. Although sequence chimeras can now be reliably removed computationally [13], [19], [35], this filtering process is still an essential early step in any microbiome analysis.

A final consideration in the computational analysis of community structure assays is the use of microarray-based methods for 16S (and other marker) quantification within a microbiome. Just as high-throughput RNA sequencing parallels gene expression microarrays, 16S rDNA sequencing parallels phylochips, microarrays constructed with probes complementary to a variety of 16S and other marker sequences [36]. The design and analysis of such arrays can be challenging, as 16S sequences (or any good genomic markers) will be highly similar, and the potential for extensive cross-hybridization must be taken into account both when determining what sequences to place on a chip and how to quantify their abundance after hybridization [37]. The continued usefulness of such arrays will be dictated by future trends in high-throughput sequencing costs and barcoding, but at present phylochips are beginning to be constructed to capture functional sequences in combination with measures of taxon abundances in high throughput, and they represent an interesting option for population-level microbiome assays.

## 4. Shotgun Sequencing and Metagenomics

While measures of community diversity have dominated historical analyses, modern high-throughput methods are being developed for a host of other “meta” assays from uncultured microbes. The term metagenomics is used with some frequency to describe the entire body of high-throughput studies now possible with microbial communities, although it also refers more specifically to whole-metagenome shotgun (WMS) sequencing of genomic DNA fragments from a community's metagenome [38], [39]. Metatranscriptomics, a close relative, implies shotgun sequencing of reverse-transcribed RNA transcripts [40], [41], metaproteomics [42], [43] the quantification of protein or peptide levels, and metametabolomics (or less awkwardly community metabolomics) [44], [45] the investigation of small-molecule metabolites. Of these assays, the latter three in particular are still in their infancy, but are carried out using roughly the same technologies as their culture-based counterparts, and the resulting data can typically be analyzed using comparable computational methods.

As of this writing, no complete metametabolomic studies from uncultured microbiomes have yet been published, although their potential usefulness in understanding e.g. the human gut microbiome and its role in energy harvest, obesity, and metabolic disorders is clear [44]. Metaproteomic and metatranscriptomic studies have primarily focused on environmental samples [46], [47], [48], but human stool metatranscriptomics [41], [49] and medium-throughput human gut metaproteomics [42], [43] have also been successfully executed and analyzed using bioinformatics similar to those for metagenomes (see below) [42]. Quantification of the human stool metatranscriptome and metaproteome in tandem with host biomolecular activities should yield fascinating insights into our relationship with our microbial majority.

DNA extraction and WMS sequencing from uncultured samples developed, like many sequencing technologies, concurrently with the Human Genome Project [2], [50], [51], [52], and as with other community genomic assays, the earliest applications were to environmental microbes due to the ease of isolation and extraction [53], [54]. WMS techniques are in some ways much the same now as they were then, modulo the need for complex Sanger clone library construction: isolate microbial cells of a target size range (e.g. viral, bacterial, or eukaryotic), lyse the cells (taking care not to lose DNA to native DNAses), isolate DNA, fragment it to a target length, and sequence the resulting fragments [55], [56]. Since this procedure can be performed on essentially any heterogeneous population, does not suffer from the single-copy and evolutionary assumptions of marker genes, and does not require (although can include) amplification, it can to some degree produce a less biased community profile than does 16S sequencing [57].

### 4.1 Metagenome Data Analysis

Unlike whole-genome shotgun (WGS) sequencing of individual organisms, in which the end product is typically a single fully assembled genome, metagenomes tend not to have a single “finish line” and have been successfully analyzed using a range of assembly techniques. The simplest is no assembly at all - the short reads produced as primary data can, after cleaning to reduce sequencing error [18], be treated as taxonomic markers or as gene fragments and analyzed directly. Since microbial genomes typically contain few intergenic sequences, most fragments will contain pieces of one or more genes; these can be used to quantify enzymatic or pathway abundances directly as described below [1], [58], [59], [60]. Alternatively, metagenome-specific assembly algorithms have been proposed that reconstruct only the open reading frames from a population (its ORFeome), recruiting highly sequence-similar fragments on an as-needed basis to complete single gene sequences and avoiding assembly of larger contigs [61], [62]. The most challenging option is to attempt full assemblies for complete genomes present in the community, which is rarely possible save in very simple communities or with extreme sequencing depth [53], [54]. When successful, this has the obvious benefit of establishing synteny, structural variation, and opening up the range of tools developed for whole-genome analysis [63], and guided assemblies using read mapping (rather than *de novo* assembly) can be used when appropriate reference genomes are available. However, care must be taken in interpreting any such assemblies, since horizontal transfer and community complexity prevent unambiguous assemblies in essentially all realistic cases [64]. A more feasible middle ground is emerging around maximal assemblies that capture the largest unambiguous contigs in a community [65], allowing e.g. local operon structure to be studied without introducing artificial homogeneity into the data. In any of these cases - direct analysis of reads, ORF assembly, maximal unambiguous scaffolds, or whole genomes - subsequent analyses typically focus on the functional aspects of the resulting genes and pathways as detailed below.

A key bioinformatic tradeoff in analyzing metagenomic WMS sequences, regardless of their degree of assembly, is whether they should be analyzed by homology, *de novo*, or a combination thereof. An illustrative example is the task of determining which parts of each sequence read (or ORF/contig/etc.) encode one or more genes, i.e. gene finding or calling. By homology, each sequence can be BLASTed [66] against a large database of reference genomes, which will retrieve any similar known reading frames; the boundaries of these regions of similarity thus become the start and stop of the metagenomic open reading frames. This method is robust to sequencing and assembly errors, but it is sensitive to the contents of the reference database. Conversely, *de novo* methods have been developed to directly bin [67], [68], [69] and call genes within [61], [62] metagenomic sequences using DNA features alone (GC content, codon usage, etc.). As with genome analysis for newly sequenced single organisms, most *de novo* methods rely on interpolated [70] or profile [71] Hidden Markov Models (HMMs) or on other machine learners that perform classification based on encoded sequence features [72], [73]. This is a far more challenging task, making it sensitive to errors in the computational prediction process, but it enables a greater range of discovery and community characterization efforts by relying less on prior knowledge. Hybrid methods for e.g. taxonomic binning [69] have recently been developed that consume both sequence similarity and *de novo* sequence features as input, and for some tasks such systems might represent a sweet spot between computational complexity, availability of prior knowledge, and biological accuracy. This tradeoff between knowledge transfer by homology and *de novo* prediction from sequence is even more pronounced when characterizing predicted genes, as discussed below.

## 5. Computational Functional Metagenomics

Essentially any analysis of a microbial community is “functional” in the sense that it aims to determine the overall phenotypic consequences of the community's composition and biomolecular activity. For example, the Human Microbiome Project began to investigate what typical human microbial community members are doing [60], how they are affecting their human hosts [2], what impact they have on health or disease, and these help to suggest how pro- or antibiotics can be used to change community behavior for the better [74]. The approaches referred to as computational functional metagenomics, however, typically focus on the function (either biochemically or phenotypically) of individual genes and gene products within a community and fall into one of two categories. Top-down approaches screen a metagenome for a functional class of interest, e.g. a particular enzyme family, transporter or chelator, pathway, or biological activity, essentially asking the question, “Does this community carry out this function and, if so, in what way?” Bottom-up approaches attempt to reconstruct profiles, either descriptive or predictive, of overall functionality within a community, typically relying on pathway and/or metabolic reconstructions and asking the question, “What functions are carried out by this community?”

Either approach relies, first, on cataloging some or all of the gene products present in a community and assigning them molecular functions and/or biological roles in the typical sense of protein function predictions [53], [54], [59]. As with so many bioinformatic methods, the simplest techniques rely on BLAST [66]: a top-down investigation can BLAST representatives of gene families of interest into the community metagenome to determine their presence and abundance [63], and a bottom-up approach can BLAST reads or contigs from a metagenome into a large annotated reference database such as nr to perform knowledge transfer by homology [75], [76], [77]. Top-down approaches dovetail well with experimental screens for individual gene product function [6], and bottom-up approaches are more descriptive of the community as a whole [78].

As each metagenomic sample can contain millions of reads and databases such as nr in turn contain millions of sequences, computational efficiency is a critical consideration in either approach. On one hand, stricter nucleotide searches or direct read mapping to reference genomes [79], [80] improve runtime and specificity at the cost of sensitivity; on the other, more flexible characterizations of sequence function such as HMMs [72], [73] tend to simultaneously increase coverage, accuracy, and computational expense. Any of these sequence annotation methods can be run directly on short reads, on ORF assemblies, or on assembled contigs, and statistical methods have been proposed to more accurately estimate the frequencies of functions in the underlying community when they are under-sampled (requiring the estimation of unobserved values [81]) or over-sampled (correcting for loci with greater than 1× coverage [82]). In any of these cases, the end result of such an analysis is an abundance profile for each metagenomic sample quantifying the frequency of gene products in the community; the profiles for several related communities can be assembled into a frequency matrix resembling a microarray dataset. Gene products (rows) in such a profile can be identified by functional descriptors such as Gene Ontology [83] or KEGG [84] terms, protein families such as Pfams [73] or TIGRfams [72], enzymatic [85], transport [86], or other structural classes [87], or most often as orthologous families such as HomoloGenes [88], COGs [89], NOGs [90], or KOs [84].

A logical next step, given such an abundance profile of orthologous families, is to assemble them into profiles of community metabolic and functional pathways. This requires an appropriate catalog of reference pathways such as KEGG [84], MetaCyc [91], or GO [83], although it should be noted that none of these is currently optimized for modeling communities rather than single organisms in monoculture [90]. The pathway inference process is similar to that performed when annotating an individual newly sequenced genome [92] and consists of three main steps: A) assigning each ortholog to one or more pathways, B) gap filling or interpolation of missing annotations, and C) determining the presence and/or abundance of each pathway. The first ortholog assignment step is necessary since many gene families participate in multiple pathways; phosphoenolpyruvate carboxykinase, for example, is used in the TCA cycle, glycolysis, and in various intercellular signaling mechanisms [93]. The abundance mass for each enzyme is distributed across its functions in one or more possible pathways; methods for doing this range from the simple assumption that it is equally active in all reference pathways (as currently done by KAAS [94] or MG-RAST [76]) to the elimination of unlikely pathways and the redistribution of associated mass in a maximum parsimony fashion [95]. Second, once all observed orthologs have been assigned to pathways (when possible), gaps or holes in the reference pathways can be filled, using the assumption that the enzymes necessary to operate a nearly complete pathway should be present somewhere in the community. Essentially three methods have been successfully employed for gap filling: searching for alternative pathway fragments to explain the discrepancy [96], [97], purely mathematical smoothing to replace the missing enzymes' abundances with numerically plausible values [81], and targeted searches of the metagenome of interest for more distant homologues with which to fill the hole [98]. Since we are currently able to infer function for only a fraction of the genes in any given complete genome, let alone metagenome, any of these approaches should be deemed hypothetical at best; nevertheless, like any missing value imputation process, they can provide numerically stable guesses that are substantially better than random [99]. Finally, as described above for taxa, the resulting data can be used to summarize each reference pathway either qualitatively (i.e. with what likelihood is it present in the community?) or quantitatively (how abundant is it in the community?), and in its simplest form condenses the abundance matrix of orthologous families into an abundance (or presence/absence) matrix of pathways. Either the ortholog or pathway matrices can then be tested for differentially abundant features representing diagnostic biomarkers with potential explanatory power for the phenotype of interest, using statistical methods developed for identical tests in expression biomarker discovery [100] and genome-wide association studies [101].

However, our prior knowledge of (primarily) metabolic pathways can be leveraged to produce richer inferences from such pathway abundance information. Given sufficient information about the pathways in a community, it is relatively straightforward to predict what metabolic compounds have the potential to be produced. However, it is much more difficult to infer what metabolite pools and fluxes in the community will actually be under a specific set of environmental conditions [102], [103]. Multi-organism flux balance analysis (FBA) is an emerging tool to enable such analyses [104], but given the extreme difficulty of constructing accurate models for even single organisms [105] or of determining model parameters in a multi-organism community [53], no successful reconstructions have yet been performed for complex microbiomes. The area holds tremendous promise, however, first with respect to metabolic engineering - it is not yet clear what successes might be achieved with respect to biofuel production or bioremediation using synthetically manipulated communities in place of individual organisms [106], [107]. Second, in addition to metabolite profiling, multi-organism growth prediction allows the determination of mutualisms, parasitisms, and commensalisms among taxa in the community [108] [109], [110], opening the door to basic biological discoveries regarding community dynamics [25], [111], [112] and to therapeutic probiotic treatments for dysbioses in the human microbiome [113], [114].

## 6. Host Interactions and Interventions

A final but critical aspect of translational metagenomics lies in understanding not only a microbial community but also its environment - that is, its interaction with a human host. Our microbiota would be of interest to basic research alone if they were not heavily influenced by host immunity and, in turn, a major influence on host health and disease. The skin of humans hosts relatively few taxa (e.g. *Propionibacterium* [115]), the nasal cavity somewhat more (e.g. *Corynebacterium* [116]), the oral cavity (dominated by *Streptococcus*) several hundred taxa (with remarkable diversity even among saliva, tongue, teeth, and other substrates [117], [118]) and the gut over 500 taxa with densities over 10^11^ cells/g [2], [119]. Almost none of these communities are yet well-understood, although anecdotes abound. The skin microbiome is thought to be a key factor in antibiotic resistant *Staphylococcus aureus* infections [120], [121]; nasal communities have interacted with the pneumococcus population to influence its epidemiological carriage patterns subsequent to vaccination programs [122]; and extreme dysbiosis in cystic fibrosis can be a precursor to pathogenic infection [123].

The gut, however, is currently the best-studied human microbiome [119], [124], [125]. It is a dynamic community changing over the course of days [126], [127], over the longer time scales of infant development [112], [128], [129], [130] and aging [131], [132], in response to natural perturbations such as diet [59], [133], [134], [135] and illness [114], [136], and modified in as-yet-unknown ways by the modern prevalence of travel, chemical additives, and antibiotics [126]. Indeed, the human gut microbiome has proven difficult to study exactly because it is so intimately related to the physiology of its host; inasmuch as no two people share identical microbiota, most microbiomes are strikingly divergent between distinct host species, rendering results from model organisms difficult to interpret [137], [138]. Nevertheless, studies in wild type vertebrates such as mice [139], [140] and zebrafish [141], [142] have found a number of similarities in their microbiotic function and host interactions. In particular, germ-free organisms have yielded insights into the microbiota's role in maturation of the host immune system and, surprisingly, even anatomical development of the intestine [143], [144]. Similarly, gnotobiotic systems in which an organism's natural microbiota are replaced with their human analog are a current growth area for closer study of the phenotypic consequences of controlled microbiotic perturbations [145].

One of the highest-profile demonstrations of this technique and of the microbiota's influence on human health has been in an ongoing study of the microbiome in obesity [146]. Early studies in wild-type mice [139] demonstrated gross taxonomic shifts in the composition and diversity of the microbiomes of obese individuals; follow-ups in gnotobiotic mice confirmed that this phenotype was transmissible via the microbiome [147]. These initial studies were taxonomically focused and found that, while high-level phyla were robustly perturbed in obesity (which incurs a reduction in *Bacteroidetes* and concomitant increase in *Firmicutes* [139]), few if any specific taxa seemed to be similarly correlated [138], [140]. Subsequent functional metagenomics, first in mouse [148] and later a small human cohort [59], established that the functional consistency of these shifts operates more consistently, enriching the microbiome's capacity for energy harvest and disregulating fat storage and signaling within the host. While these observations represent major descriptive triumphs, further computational and experimental work must yet be performed to establish the underlying biomolecular mechanisms and whether they are correlative, causative, or may be targeted by interventions to actively treat obesity [59].

A similarly complex community for which we have a greater understanding of the functional mechanisms at play is the formation of biofilms in the oral cavity preceding caries (cavities) or periodontitis [149]. While we are still investigating the microbiota of the saliva [150] and of the oral soft tissues [151], colonization of the tooth enamel is somewhat better understood due to the removal of significant interaction with host tissue. Even more strikingly, this biofilm, or physically structured consortium of multiple microbial taxa, must reestablish itself from almost nothing each time we brush our teeth - a process that can be achieved within hours [152]. Streptococci in particular possess a number of surface adhesins and receptors that enable them to behave as early colonizers on bare tooth surface and to bind together a variety of subsequent microbes [153]. These fairly minimal bacteria are metabolically supported by *Veillonella* and *Actinomyces* species, and their aggregation leads to local nutritive and structural environments favorable to e.g. *Fusobacterium* and *Porphyromonas* [154]. Each of these steps is mediated by a combination of cell surface recognition molecules, extracellular physical interactions, metabolic codependencies, and explicit intercellular signaling, providing an excellent example of the complexity with which structured microbiomes can evolve. Indeed, the evolvability of such systems, both as a whole [155] and at the molecular level [156], is yet another aspect of the work remaining to computationally characterize microbiotic biomolecular and community function.

Finally, the microbiota clearly represent a key component of future personalized medicine. First, the number and diversity of phenotypes linked to the composition of the microbiota is immense: obesity, diabetes, allergies, autism, inflammatory bowel disease, fibromyalgia, cardiac function, various cancers, and depression have all been reported to correlate with microbiome function [157]. Even without causative or modulatory roles, there is tremendous potential in the ability to use the taxonomic or metagenomic composition of a subject's gut or oral flora (both easily sampled) as a diagnostic or prognostic biomarker for any or all of these conditions. Commercial personal genomics services such as 23andMe (Mountain View, CA) promise to decode your disease risk based on somatic DNA from a saliva sample; bioinformatic techniques have yet to be developed that will allow us to do the same using microbial DNA.

Second, the microbiota are amazingly plastic; they change metagenomically within hours and metatranscriptomically within minutes in response to perturbations ranging from broad-spectrum antibiotics to your breakfast bacon and eggs [41], [126], [127]. For any phenotype to which they are causally linked, this opens the possibility of pharmaceutical, prebiotic (nutrients promoting the growth of beneficial microbes [113], [119]), or probiotic treatments. Indeed, Nobel Prize winner Ilya Mechnikov famously named *Lactobacillus bulgaricus*, a primary yogurt-producing bacterium, for its apparent contribution to the longevity of yogurt-consuming Bulgarians [158], and despite a degree of unfortunate popular hype, the potential health benefits of a variety of probiotic organisms are indeed supported by recent findings [125], [159]. Unfortunately, we currently understand few of the mechanisms by which these interventions operate. Do the supplemented organisms outcompete specific pathogens, do they simply increase their own numbers, or do they shift the overall systems-level balance of many taxa within the community? Do they reduce the levels of detrimental metabolites in the host, or do they increase the levels of beneficial compounds? Do they change biomolecular activity being carried out in microbial cells, adjacent host epithelial or immune cells, or distal cells through host signaling mechanisms? Or, as in polygenic genetic disorders, does a combination of many factors result in health or disease status as an emergent phenotype?

The human microbiome has been referred to as a “forgotten organ” [160], and the truth of both words is striking. Our trillions of microbial passengers account for a proportion of our metabolism and signaling as least as great as that performed by more integral body parts, and after a century of molecular biology, we have only begun to realize their importance within the last few years. To close with a success story, the popular press [161] recently reported on the full recovery of a patient suffering from *Clostridium difficile*-associated diarrhea, which had led her to lose over 60 pounds in less than a year. *C. difficile* is often refractory to antibiotics, with spores able to repopulate from very low levels, and the patient's normal microbiota had been decimated by the infection and subsequent treatment. Finally, she received a simple fecal transplant from her husband, in which the host microbiome was replaced with that of a donor. Within days, not only had she begun a complete recovery, but a metagenomic survey of her microbiota showed that the new community was almost completely established and had restored normal taxonomic abundances [162]. While this is an extreme case, similar treatments have shown a success rate of some 90% historically [163], all of which occurred before modern genomic techniques allowed us to more closely examine the microbiota. Imagine performing any other organ transplant with such a high rate of success - while blindfolded! Like so many other discoveries of the genomic era, the study of the human microbiome has begun with amazing achievements, and it will require continued experimental and bioinformatic efforts to better understand the biology of these microbial communities and to see it translated into clinical practice.

## 7. Summary

The human microbiome consists of unicellular microbes - mainly bacterial, but also archaeal, viral, and eukaryotic - that occupy nearly every surface of our bodies and have been linked to a wide range of phenotypes in health and disease. High-throughput assays have offered the first comprehensive culture-free techniques for surveying the members of these communities and their biomolecular activities at the transcript, protein, and metabolic levels. Most current technologies rely on DNA sequencing to examine either individual taxonomic markers in a microbial community, typically the 16S ribosomal subunit gene, or the composite metagenome of the entire community. Taxonomic analyses lend themselves to computational techniques rooted in microbial ecology, including diversity measures within (alpha) and between (beta) samples; these can be defined quantitatively (based on abundance) or qualitatively (based on presence/absence), and they may or may not take into account the phylogenetic relatedness of the taxa being investigated. Finally, in the absence of information regarding specific named species in a community, sequences are often clustered by similarity into Operational Taxonomic Units (OTUs) as the fundamental unit of analysis within a sample.

In contrast, whole-genome shotgun analyses begin with sequences sampled from the entire community metagenome. These can also be taxonomically binned, or they can be assembled, partially assembled into ORFeomes, or characterized directly at the read level. Characterization typically consists of function assignment similar to that performed for genes during annotation of a single organism's genome; once genes in the metagenome are defined, they can be mapped or BLASTed to reference sequence databases or analyzed intrinsically using e.g. codon frequencies or HMM profiles. Finally, the frequencies of enzymes and other gene products so determined can be assigned to pathways, allowing inference of the overall metabolic potential of the community and inference of diagnostic and potentially explanatory functional biomarkers. Ongoing studies are beginning to investigate the ways in which the microbiota can be directly engineered using pharmaceuticals, prebiotics, probiotics, or diet as a preventative or treatment for a wide range of disorders.

## 8. Exercises

Q1. You have a collection of 16S rRNA gene sequencing data, which consists of an Illumina run in which the 100 bp V6 hypervariable region has been amplified. The error rate of Illumina sequencing has been estimated as 1.3×10^−3^ per base pair [164], and you have 30 million Illumina reads. Will binning your reads into OTUs at 100% or 97% give you a more interpretable estimation of the number of OTUs present? Why?

Q2. You have collections of 16S rRNA gene reads from two environmental samples, A and B. You examine 50 reads each from sample A and sample B, which correspond to four taxa in A and two taxa in B. You examine 25 more reads from each library and detect two more taxa in A and one more in B. In total, two of these taxa are present in both communities A and B. Which sample has higher alpha diversity by counting taxonomic richness? What is the beta diversity between A and B using simple overlap of taxa? Using Bray-Curtis dissimilarity?

Q3. You examine 1,000 more sequences from samples A and B, detecting 10 additional taxa in A and 25 in B. Which sample has higher alpha diversity now, as measured by taxonomic richness? Why is this different from your previous answer? What statement can you make about the ecological evenness of communities A and B as a result?

Q4. What factors in the microbial environment might you expect to be reflected in metabolism, signaling, and biomolecular function between skin bacteria and oral bacteria? What impact would you expect this to have on the pathways carried in these community metagenomes, or on their alpha diversities?

Q5. It is estimated that 2–5% of the population has *Clostridium difficile* in their intestines. Why is this not usually a problem?

Q6. Consider the impact upon the human microbiome of two perturbations: social contact and brushing your teeth. What short-term and long-term impact do you expect on alpha diversity? Beta diversity?

Q7. Calculate richness, the inverse Simpson index, and the Shannon index for each sample described in the table below. Which has the highest alpha diversity? Why is the answer different according to which measurement you use?



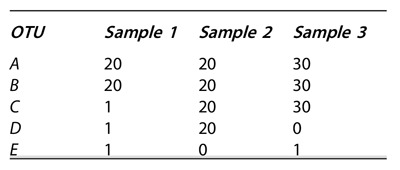



Answers to the Exercises can be found in Text S1.

**Further Reading**

It is difficult to recommend comprehensive literature in an area that is changing so rapidly, but the bioinformatics of microbial community studies are currently best covered by the reviews in [22], [56], [165]. Computational tools for metagenomic analysis include [13], [19], [63], [75], [76], [77], [166]. An overview of microbial ecology from a phylogenetic perspective is provided in [167], [168], and the use of the 16S subunit as a marker gene is reviewed in [12]. Likewise, experimental and computational functional metagenomics are discussed in [6], [25], [169]. The clinical relevance of the human microbiome is far-ranging and is comprehensively reviewed in [157].

**Glossary**

alpha diversity: within-sample taxonomic diversity

beta diversity: between-sample taxonomic diversity

binning: assignment of sequences to taxonomic units

biofilm: a physically (and often temporally) structured aggregate of microorganisms, often containing multiple taxa, and often adhered to each other and/or to a defined substrate

chimera: an artificial DNA sequence generated during amplification, consisting of a combination of two (or more) true underlying sequences

collector's curve: a plot in which the horizontal axis represents samples (often DNA sequences) and the vertical axis represents diversity (e.g. number of distinct taxa)

community structure: used most commonly to refer to the taxonomic composition of a microbial community; can also refer to the spatiotemporal distribution of taxa

diversity: a measure of the taxonomic distribution within a community, either in terms of distinct taxa or in terms of their evolutionary/phylogenetic distance

FBA: Flux Balance Analysis, a computational method for inferring the metabolic behavior of a system given prior knowledge of the enzymatic reactions of which it is capable

functional metagenomics: computational or experimental analysis of a microbial community with respect to the biochemical and other biomolecular activities encoded by its composite genome

gap filling: the process of imputing missing or inaccurate gene abundances in a set of pathways

germ-free: a host animal containing no microorganisms

gnotobiotic: a host animal containing a defined set of microorganisms, either synthetically implanted or transferred from another host; often used to refer to model organisms with humanized microbiota

holes: missing genes in a set of reference pathways; see gap filling

interpolation: see gap filling

marker: a gene or other DNA sequence that can be (ideally) unambiguously assigned to a particular taxon or function

metagenome: the total genomic DNA of all organisms within a community

metagenomics: the study of uncultured microbial communities, typically relying on high-throughput experimental data and bioinformatic techniques

metametabolome: the total metabolite pool (and possibly fluxes) of a community

metaproteome: the total proteome of all organisms within a community

metatranscriptome: the total transcribed RNA pool of all organisms within a community

microbiome: the total microbial community and biomolecules within a defined environment

microbiota: the total collection of microbial organisms within a community, typically used in reference to an animal host

microflora: an older term used synonymously with microbiota

ORFeome: the total collection of open reading frames within a metagenome

ortholog: in strict usage, a homologous gene in two species distinguished only by a speciation event; in practice, used to denote any gene sufficiently homologous as to represent strong evidence for conserved biological function

OTU: Operational Taxonomic Unit, a cluster of organisms similar at the sequence level beyond some threshhold (e.g. 95%) used in place of species, genus, etc.

phylochip: a microarray containing taxonomic (and sometimes functional) marker sequences

phylotype: see OTU

prebiotic: a food substance metabolized by the microbiota so as to directly or indirectly benefit the host

probiotic: a live microorganism consumed by the host with direct or indirect health benefits

rarefaction curve: see collector's curve

richness: see diversity

16S rRNA: the transcribed form of the 16S ribosomal subunit gene, the smaller RNA component of the prokaryotic ribosome, used as the most common taxonomic marker for microbial communities

WGS: Whole-Genome Shotgun, used to describe shotgun sequencing of individual organisms and, sometimes, microbial communities, although this is not completely accurate as no “whole-genome” is typically involved

WMS: Whole-Metagenome Shotgun sequencing, used in reference to undirected metagenomic sequencing to distinguish it from sequencing directed to specific taxonomic marker genes

## Supporting Information

Text S1 Answers to Exercises.
(DOCX)
