# Supplementary material for: Chapter 12: Human Microbiome Analysis
Source: PLoS Comput Biol. 2012 Dec 27;8(12):e1002808. doi: 10.1371/journal.pcbi.1002808 (PMC3531975; doi:10.1371/journal.pcbi.1002808)
Supplement: Text S1 — Answers to Exercises. (DOCX) [file pcbi.1002808.s001.docx]

# Exercises and Answers

Q1. You have a collection of 16S rRNA gene sequencing data, which consists of an Illumina run in which the 100 bp V6 hypervariable region has been amplified. The error rate of Illumina sequencing has been estimated as 1.3 x 10^-3^ per base pair [[169](#_ENREF_169)], and you have 30 million Illumina reads. Will binning your reads into OTUs at 100% or 97% give you a more interpretable estimation of the number of OTUs present? Why?

A1. An error rate of 0.0013 means that there will be, on average, one sequencing error per ten 100 bp sequences. Thus, in a pool of 30 million reads, there will be large numbers of very similar but non-identical sequences. Binning sequences into OTUs at 100% similarity will thus grossly inflate the actual numbers of unique taxa present. Binning at 97% similarity, in contrast, will allow highly-similar but non-identical sequences to belong to the same OTU and result in a representation of 16S gene sequence families that likely better reflects those that are actually present. Note that these numbers do not take additional sources of error such as chimerism into account, and that the expected number of mutations due to evolution rather than sequencing error varies greatly depending on the length of sequence and its position within the 16S gene.

Q2. You have collections of 16S rRNA gene reads from two environmental samples, A and B. You examine 50 reads each from sample A and sample B, which correspond to four taxa in A and two taxa in B. You examine 25 more reads from each library and detect two more taxa in A and one more in B. In total, two of these taxa are present in both communities A and B. Which sample has higher alpha diversity by counting taxonomic richness? What is the beta diversity between A and B using simple overlap of taxa? Using Bray-Curtis dissimilarity?

A2. Sample A has higher alpha diversity than sample B as measured by taxonomic richness, six in A and three in B. The beta diversity between A and B as measured by taxon overlap is β = (*S*_1_ − *c*) + (*S*_2_ − *c*), or β = (6-2) + (3-2) = 4 + 1 = 5. The beta diversity as measured by the Bray-Curtis dissimilarity is ${BC}_{ij}=\frac{S_{i}+S_{j}-2C_{ij}}{S_{i}+S_{j}}$ = $\frac{6+3-2(3)}{6+3}$ = 3/9 = 0.33.

Q3. You examine 1,000 more sequences from samples A and B, detecting 10 additional taxa in A and 25 in B. Which sample has higher alpha diversity now, as measured by taxonomic richness? Why is this different from your previous answer? What statement can you make about the ecological evenness of communities A and B as a result?

A3. Sample B has higher alpha diversity than sample A. Here, there is now sufficient sequencing depth to detect taxa that are present at very low abundance. Sample B has more biological diversity than Sample A, but many of the species are present in very small numbers, making A a more even community that might also be considered to be more diverse by measures more complex than simple richness.

Q4. What factors in the microbial environment might you expect to be reflected in metabolism, signaling, and biomolecular function between skin bacteria and oral bacteria?  What impact would you expect this to have on the pathways carried in these community metagenomes, or on their alpha diversities?

A4. There are many possible answers to this question. The pH of skin is around 5.5-5.9, while mouth pH ranges from 5.5-7 but is also capable of forming highly acidic microenvironments. In addition, most skin surfaces are constantly exposed to air with limited microanerobic environments, while high and low-oxygen environments are both prevalent in the mouth. Skin bacteria will use primarily oil and dead skin as nutrient sources, while mouth bacteria will have limited access to whatever the host has eaten. We would therefore expect oral bacterial communities to contain a broader range of pH tolerance mechanisms, both aerobic and anaerobic metabolism, and a wide variety of enzymes for rapidly breaking down carbohydrates (particularly simple sugars) when compared to skin bacterial communities. The mouth would be expected to have a higher level of alpha diversity due to the wide variety of microenvironments, whereas the skin might have higher beta diversity due to the ease with which it can be disrupted on different individuals in different environments.

Q5. It is estimated that 2-5% of the population has *Clostridium difficile* in their intestines. Why is this not usually a problem?

A5. Under normal circumstances, native gut bacteria will out-compete *C. difficile*, and it will be controlled by host immunity. Its population does not typically become overgrown unless the native gut bacteria are severely disrupted. In practice, this phenotype is almost always due to the use of broad-spectrum antibiotics.

Q6. Consider the impact upon the human microbiome of two perturbations: social contact and brushing your teeth. What short-term and long-term impact do you expect on alpha diversity? Beta diversity?

A6. Social contact will increase exposure to more types of microbes, and will likely increase alpha diversity both short-term and long-term. It will likewise increase beta diversity on the skin as different individuals encounter different environments. Brushing teeth will cause a short-term drop in diversity as a large bacterial population is killed and goes through a bottleneck, but repeated perturbation will create a wider variety of environmental niches and will thus likely increase diversity in the long run. It might not have a strong effect on beta diversity, as it will not provide a substantially different environment or microbial exposure among individuals.

Q7. Calculate richness, the inverse Simpson index, and the Shannon index for each sample described in the table below. Which has the highest alpha diversity? Why is the answer different according to which measurement you use?

$$\begin{matrix} OTU \\ A \\ B \\ C \\ D \\ E \end{matrix} \begin{matrix} Sample 1 & Sample 2 & Sample 3 \\ 20 & 20 & 30 \\ 20 & 20 & 30 \\ 1 & 20 & 30 \\ 1 & 20 & 0 \\ 1 & 0 & 1 \end{matrix}$$

A7.

Sample 1: Richness = 5, inv. Simpson = 2.4, Shannon (log) = 1.4.

Sample 2: Richness = 4, inv. Simpson = 4.2, Shannon (log) = 2.

Sample 3: Richness = 4, inv. Simpson = 3.2, Shannon (log) = 1.66

The Sampson and Shannon indices are influenced by the evenness of taxon distribution, whereas richness is not.
